# Recent Progress on Patterning Strategies for Perovskite Light‐Emitting Diodes toward a Full‐Color Display Prototype

**DOI:** 10.1002/smsc.202000050

**Published:** 2021-02-03

**Authors:** Yatao Zou, Lei Cai, Tao Song, Baoquan Sun

**Affiliations:** ^1^ Jiangsu Key Laboratory for Carbon-Based Functional Materials and Devices Institute of Functional Nano and Soft Materials (FUNSOM) Joint International Research Laboratory of Carbon-Based Functional Materials and Devices Soochow University 199 Ren'ai Road Suzhou Jiangsu 215123 P. R. China

**Keywords:** high-resolution perovskite microarrays, patterned metal halide perovskite, perovskite emitters, perovskite light-emitting diodes

## Abstract

Perovskite light‐emitting diodes (PeLEDs) have attracted both academic and industrial interest because of their high efficiency, wide color gamut as well as low material and fabrication costs. However, most state‐of‐the‐art PeLEDs are still fabricated with commonly used spin‐coating methods, which are undesirable for large‐scale commercial production. Achieving highly emissive perovskite microarrays at high spatial resolution with quick and large‐scale growth is one of the critical steps to integrate state‐of‐the‐art PeLEDs into full‐color display panels. Here, the fabrication methods and crystallization processes of perovskite materials are first discussed because they are strongly relevant to the patterning process and the quality of the perovskite pixels. Then, the current strategies to realize perovskite patterns that can be likely integrated into display panels, through mask‐free or mask‐assisted methods, are explored. Self‐emitting and down‐conversion PeLED devices with a patterned perovskite matrix as the emissive layer are also reviewed. Finally, an outlook is provided on how to further improve the optical and electrical properties of the perovskite patterns and the performance of PeLEDs as well as to develop eco‐friendly devices to accelerate the potential commercialization progress of this young technique.

## Graphical abstract

Achieving highly emissive perovskite microarrays at high spatial resolution with quick and large‐scale growth is one of the critical steps to integrate state‐of‐the‐art perovskite light‐emitting diodes (PeLEDs) into full‐color display panels. The recent progress on patterning PeLEDs is reviewed and an outlook is provided on the future direction to promote the potential commercialization progress of PeLEDs.

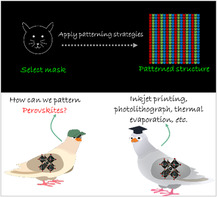

## 1 Introduction

Perovskite light‐emitting diodes (PeLEDs) are a rising technique and have attracted a lot of interest in both academic and industrial fields because of their high efficiency, wide color gamut, and low material as well as fabrication costs.^[^ ^1^ ^]^ The active layers in PeLEDs are based on a big class of metal halide perovskite (MHP) semiconductors, which have a general formula of ABX_3_, where A, B, and X are monovalent cations (i.e., Cs^+^, MA^+^, and FA^+^), divalent cations (i.e., Sn^2+^ and Pb^2+^), and halide ions (i.e., Cl^−^, Br^−^, and I^−^),^[^ ^2^ ^]^ respectively. The narrow emission width of MHPs makes the Commission Internationale de l'Éclairage coordinates of PeLEDs reach 140% of the National Television System Committee color standard,^[^ ^3^ ^]^ which is much wider than those of the well‐established liquid crystal displays (LCDs) and organic LEDs (OLEDs). Although the electroluminescence (EL) of PeLEDs at room temperature was not observed until 2014 by Tan et al.,^[1a]^ the external quantum efficiencies (EQEs) of blue, green, and red emission have been dramatically increased to 12.3%,^[^ ^4^ ^]^ 22%,^[^ ^4^ ^]^ and 21.3%^[^ ^5^ ^]^ with great effort made on engineering the perovskite composition and device structure in the past few years, exhibiting huge potential for industrial applications. Although LCDs and OLEDs are major techniques in display markets at present, the limited color gamut of LCDs and relatively short life span (always shorter than 4 years due to the thermal and/or humidity instability of the organic materials) as well as high material costs of OLEDs still leave enough space for the development of next‐generation candidates based on MHPs, such as mini‐LEDs and micro‐LEDs.^[^ ^6^ ^]^

The manufacturing of display panels in particular needs to pattern the red–green–blue emitters side by side at high spatial resolution.^[^ ^7^ ^]^ However, current studies in the PeLED community mainly focus on engineering the device structure,^[^ ^8^ ^]^ tailoring the perovskite film composition,[^1^, ^2^, ^9^] and getting depth insight into the photophysics^[^ ^10^ ^]^ to improve their EL performance. Investigations on patterned MHPs with high resolution and photoluminescence quantum efficiency (PLQE) for full‐color displays are still lacking but will be important for their future practical applications. The commonly used spin‐coating methods remain a big gap to bring the state‐of‐the‐art PeLEDs from the laboratory into commercial information displays due to their inherent drawbacks. For example, over 90% of the functional solutions will be wasted during the spin‐coating process, dramatically raising the material costs. Moreover, it also sets obstacles for making compact multicolor (including blue, green, and red) perovskite patterns side by side on the same substrate, which is a prerequisite for the industrial production of full‐color display panels. In addition to improving the efficiencies of PeLEDs, therefore, developing rational strategies to produce MHP patterns with high spatial resolution, homogeneity, and throughput as well as decent optical properties is equally critical to promote the practical applications of PeLEDs.

**Figure 1:**
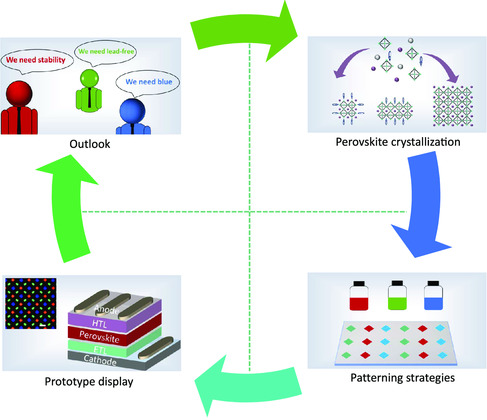
An overview of the discussion in this review.

In this Review, several patterning methods for perovskite materials and their potential applications in full‐color PeLEDs displays are reviewed. As an overview provided in **Figure** **1**, we first discuss the deposition methods and the crystallization process of MHPs because they significantly determine the following patterning process and the quality of the perovskite pixels. Then, current studies on the fabrication of patterned MHP emitters with mask‐free (i.e., jet printing and laser printing) and mask‐assisted (i.e., nanoimprinting, photolithograph, and vacuum thermal evaporation) methods are reviewed. We also discuss the current attempts to integrate these patterned MHP emitters into full‐color displays, including self‐emitting and down‐conversion PeLEDs. Finally, we provide an overview on further improving the optical and electrical properties of perovskite patterns as well as the performance of PeLEDs and developing eco‐friendly devices to accelerate the commercialization steps of this celebrated technique.

## 2 Brief Introduction of PeLEDs

**Figure 2:**
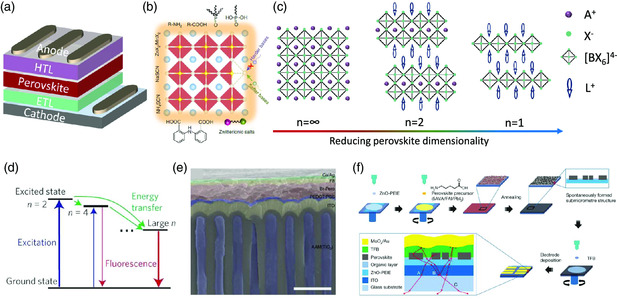
A brief introduction of PeLEDs. a) Schematic diagram of PeLEDs with multilayer architecture. b) Categories of passivating ligands for perovskite films. Reproduced with permission.^[^ ^108^ ^]^ Copyright 2020, Springer Nature. c) Perovskite structural dimensionality. d) Schematic illustration of an energy cascade in quasi‐2D perovskite films. Reproduced with permission.^[^ ^21^ ^]^ Copyright 2016, Springer Nature. e) Cross‐sectional scanning electron microscopy (SEM) image of a PeLED with a nanophotonic substrate. Reproduced under the terms of the CC‐BY 4.0 license.^[^ ^25^ ^]^ Copyright 2019, The Authors, published by Springer Nature. f) 5‐AVA is used to passivate defects in perovskite films and spontaneously form perovskite with a submicrometer‐scale structure to enhance the light outcoupling of PeLEDs. Reproduced with permission.^[1c]^ Copyright 2018, Springer Nature.

The first observation of EL from MHPs in a low‐temperature environment dates back to 1994,^[^ ^11^ ^]^ while a room temperature one was not reported until 2014 by Tan et al.^[1a]^ In a typical PeLED, the perovskite emissive layers were usually sandwiched between a hole transport layer (HTL) and an electron transport layer (ETL), as shown in **Figure** **2**a. The EQE of a PeLED is determined by the following equation

(1) $$\text{EQE}=\eta_{\text{inj}}\cdot \eta_{\text{rad}}\cdot \eta_{\text{outcoupling}}$$

where *η*_inj_, *η*_rad_, and *η*_outcoupling_ are the charge carrier injection efficiency, radiative recombination efficiency, and light outcoupling efficiency, respectively. To improve the performance of PeLEDs, numerous strategies have been used according to the three aforementioned factors. For instance, by introducing interfacial layers between perovskite layers and charge transport layers, such as polyethylenimine ethoxylated (PEIE) on the ETL of ZnO,^[8a]^ poly[(9,9‐bis(3′‐(*N*,*N*‐dimethylamion)propyl)‐2,7‐fluorene)‐alt‐2,7‐(9,9‐dioctyl)‐fluorene] (PFN) on the HTL of poly(4‐butylphenyl‐diphenyl‐amine) (poly‐TPD),^[^ ^12^ ^]^ tetraﬂuoroethylene‐perﬂuoro‐3,6‐dioxa‐4‐methyl‐7‐octene‐sulfonic acid copolymer (PFI) in poly(3,4‐ethylene dioxythiophene):poly(styrene sulfonate) (PEDOT:PSS),^[^ ^13^ ^]^ and developing multilayers of HTL[^8^, ^14^] to lower the charge carrier injection barriers, improved *η*_inj_ could be achieved. In addition, these interfacial layers were also reported to reduce the trap states of the perovskite films by passivating the surface dangling bond and/or modulating the perovskite film crystallization.^[^ ^12^, ^15^ ^]^ One may also note that the imbalanced charge injection in PeLEDs will cause the accumulation of excess charge at the perovskite interfaces, which also deteriorates the performance of PeLEDs.[^1^, ^16^] Therefore, engineering the interfacial contacts between the perovskite and charge transport layers to achieve a balanced charge injection is crucial for efficient PeLEDs.

The current methods implemented to improve the radiative recombination efficiency normally rely on two strategies, which are reducing the nonradiative recombination loss and increasing the radiative recombination rate. For the former one, the poor morphology of perovskite films is one of the main channels of nonradiative recombination loss because the injected carriers may directly recombine at the pinholes or discontinuous areas without any radiative recombination.^[^ ^17^ ^]^ Adjusting the wettability of the bottom substrates,^[^ ^12^, ^18^ ^]^ introducing antisolvent engineering during the film deposition,^[^ ^2^, ^19^ ^]^ and tailoring the perovskite composition have been substantially demonstrated to obtain compact and pinhole‐free perovskite emissive layers, which improves the performance of PeLEDs. For example, Lee and co‐workers reported a nanocrystal pinning strategy to obtain compact perovskite films, which leads to the fabrication of green PeLEDs with a peak EQE of 8.6%.^[2b]^ In addition to the poor film morphology, the deep‐level defects, especially the halide vacancies, in perovskite films and/or surfaces are other nonradiative recombination loss channels that limit the performance of PeLEDs. Thanks to the numerous theoretical and experimental investigations,^[^ ^20^ ^]^ these defects can be significantly inhibited by incorporating additives (Figure 2b), in particular those of Lewis bases and metal halide salts, which then delivers high‐performance PeLEDs. In contrast to 3D perovskites, low‐dimensional counterparts with the formula L_2_A_*n*−1_B_*n*_X_3*n*+1_ (L^+^ is a cation with lager ionic size and *n* is the number of metal halide octahedral layers between the two L^+^ cations) and perovskite quantum dots were also developed to improve the radiative recombination efficiency (Figure 2c), which was attributed to the increased radiative recombination rate due to the enhanced quantum and/or dielectric confinement effects.^[^ ^9^, ^21^ ^]^ Specifically, for quasi‐2D perovskite films, an energy cascade formed among the perovskite phases with different *n* is essential to increase the radiative recombination efficiency within the lowest‐bandgap perovskite phase (Figure 2d).

In the case of *η*_outcoupling_, previous studies have revealed it is usually below 30% in PeLEDs due to the distinct difference in the refractive index between perovskite films and other functional layers.^[^ ^22^ ^]^ A straightforward way to improve the *η*_outcoupling_ of an LED is to form periodic structures for both charge transport layers and emissive layers to reduce the total reflection of generated photons at different interfaces.^[^ ^23^ ^]^ For instance, through nanoimprinting the ETL of ZnO^[^ ^24^ ^]^ and using nanophotonic substrates^[^ ^25^ ^]^ to extract more light (Figure 2e), efficient green PeLEDs were obtained. In addition to nanoimprinting the charge transport layers and substrates, the introduction of the additives 5‐aminovaleric acid (5‐AVA) and 2,2′‐[oxybis(ethylenoxy)]diethylamine (ODEA) could passivate the defects in FAPbI_3_ films and spontaneously form submicrometer‐scale perovskite crystals as well as enhancing the light outcoupling efficiency (Figure 2f), which elevated the EQE to more than 20% for near‐infrared (NIR) PeLEDs.[^1^, ^20^] The light outcoupling efficiencies of these FAPbI_3_‐based efficient NIR devices were estimated to be over 30%.

## 3 Preparation of Perovskite Materials and Films

**Figure 3:**
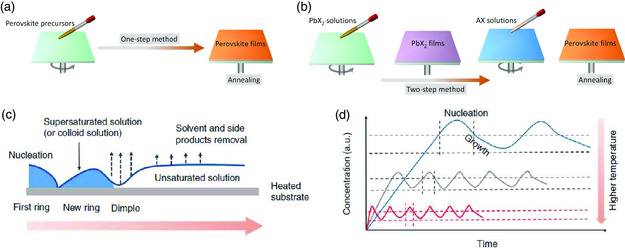
Perovskite films deposition methods and crystallization. a) The one‐step process. b) The two‐step process. c) A scheme of periodic and rhythmic crystallization. d) Schematic illustration of nucleation and growth with time based on different temperatures. c,d) Reproduced under the terms of the CC‐BY 4.0 license.^[^ ^27^ ^]^ Copyright 2017, The Authors, published by Springer Nature.

Before the exploration of any patterning strategies, we first discuss the deposition methods and crystallization processes of MHPs because they play an important role in determining the patterning process and the quality of the perovskite pixels, especially the precursor solution participating ones. Generally, there are two methods to deposit polycrystalline perovskite films, as the schematic illustration shows in **Figure** **3**a,b. For the one‐step route, a precursor solution consisting of full elements in dimethylformamide (DMF) or dimethyl sulfoxide (DMSO) is prepared and deposited on the target substrates, followed by an annealing process to promote crystallization. In terms of the two‐step one, a lead halide film is first prepared using spin coating or the vacuum thermal evaporation method, either of which is followed by treatment with halide salts (i.e., methylammonium iodide (MAI) and formamidinium iodide (FAI)) to trigger the formation of crystalline perovskites. In spite of it being a complicated process, previous studies on perovskite photovoltaics claimed that the two‐step way usually enables a smooth and compact perovskite film with less nonradiative recombination centers^[^ ^26^ ^]^ due to the readily controlled crystallization kinetics through tuning the halide salt concentration and halide salt treating time.

When depositing perovskite films on a substrate, the crystallization behavior of perovskites is decided by the supersaturation degree of the perovskite solutions, the interfacial energies, the crystallization temperature, and the wettability of the bottom substrates.^[^ ^18^, ^19^, ^27^, ^28^ ^]^ Specifically, when the solvents start to evaporate, the concentration of the solutes will increase and form a supersaturation.^[^ ^29^ ^]^ Once the solution on the substrates supersaturates, perovskite nuclei will be formed, followed by perovskite crystal growth and crystallization with further evaporation of the residual solvents in the precursor films (Figure 3c). The nucleation and growth of perovskite materials during spin coating is strongly related to the temperature of the substrates, as shown in Figure 3d. The crystallization kinetics of solution‐processed perovskite films have been thoroughly investigated by many advanced techniques, such as in situ grazing incidence wide‐angle X‐ray scattering, during both spin coating and post‐thermal‐annealing steps.^[^ ^30^ ^]^ In particular, due to the possible interaction between the metal ions and solvents (and/or the introduced additives), it was demonstrated that intermediate complexes (i.e., FAI–PbI_2_–DMF) are likely to be generated in the precursor films,^[^ ^31^ ^]^ significantly influencing the nucleation and growth of perovskite films in the following annealing steps. These intermediate complexes were reported to have the potential to increase the active formation energy of the perovskites, and hence retard the crystallization process and bring about perovskite films with large crystal size as well as suppressed defects.

Benefiting from the deep understanding of the underlying mechanism of the nucleation and crystallization, a wide range of strategies were developed to control their dynamics during spin coating or the latter annealing process by incorporating additives,[^9^, ^32^] using antisolvent engineering,[^2^, ^19^, ^33^] adjusting the properties of bottom substrates,^[^ ^18^ ^]^ and controlling the thermal annealing process.^[^ ^34^ ^]^ It is believed that a homogeneous nucleation is favorable for the formation of high‐quality perovskite films with lower defects due to the formation of an intermedia phase and retarded crystallization, resulting in improved performance of perovskite‐based optoelectronics.

In addition to polycrystalline perovskite films, perovskite quantum dots, which are usually synthesized with a hot‐injection or ligand‐assisted reprecipitation method,^[^ ^35^ ^]^ are also promising emissive materials. Regardless of the different synthesis methods, perovskite quantum dots have a general formula of perovskite/surface ligand structure. The quality of the perovskite quantum dots depends on the reaction temperature,^[^ ^36^ ^]^ reaction time,^[^ ^37^ ^]^ and properties of the surface ligands.[^35^, ^38^] Through carefully controlling the aforementioned parameters, decent perovskite quantum dot solutions with high PLQE of over 90% have been achieved.^[^ ^39^ ^]^ However, it is noteworthy that an antisolvent purification process is necessary to remove the residual organic compounds in the mother solutions;^[^ ^40^ ^]^ otherwise, the morphology and electrical transporting ability of the obtained perovskite quantum dot films can be dramatically deteriorated by these organic compounds. As such, screening rational antisolvents and chelating surface ligands is equally important to maintain the unique optical properties of the as‐synthesized quantum dots during the purification process.^[^ ^40^, ^41^ ^]^ Having obtained high‐quality perovskite quantum dots, a one‐step deposition method is widely used to deposit perovskite films on a target substrate. The perovskite quantum dots can be well dispersed in aromatic and alkane solvents, such as chlorobenzene (CB), toluene, hexane, and octane. However, taking the device structures and the physicochemical properties (i.e., viscosity, vapor pressure, and thermal conductivity) of the solvents into consideration, the solvents used to deposit perovskite films should be carefully optimized to avoid any deterioration of the underlying layers proceeding with the top one and guarantee a smooth perovskite film morphology as well as high‐performance optoelectronics.^[^ ^42^ ^]^

## 4 Mask‐Free Nanostructured MHPs

**Figure 4:**
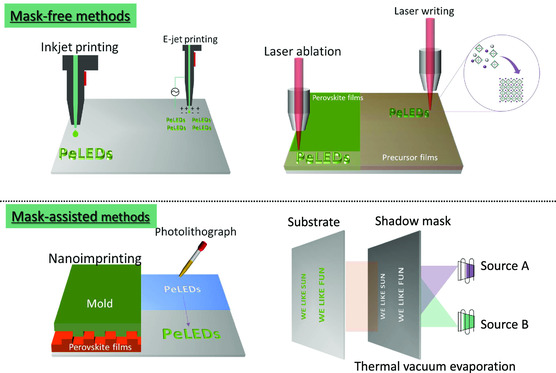
A diagram showing the representative mask‐free and mask‐assisted methods for the fabrication of perovskite patterns.

The representative methods used to pattern perovskite materials for PeLED applications are shown in **Figure** **4**. Among a number of patterning strategies, mask‐free methods provide a fast fabrication process and a noncontact strategy for patterning various materials for optoelectronics. In this section, we mainly discussed two widely used mask‐free methods of jet printing (including inkjet printing and e‐jet printing) and laser‐assisted patterning because of their potential compatibility for manufacturing high‐quality perovskite patterns that have the potential to be integrated into PeLED display panels.

### 4.1 Inkjet Printing

Inkjet printing is a well‐known technique to transfer liquid‐phase materials onto a target substrate, which relies on the generation of microdroplets (i.e., picoliter) at a nozzle aperture.^[^ ^43^ ^]^ The inkjet printing process is a well‐controlled way via a noncontact process with the application of an external signal to the print head, which provides numerous merits over other patterning methods, such as minimal waste of functional materials. In this section, regarding the different compositions of ink materials, we start by discussing the printing materials of perovskite quantum dots, which show negligible difference with the pioneer studies on other emissive materials (i.e., CdSe and organic semiconductors).[^43^, ^44^] A common concern is how to overcome the intrinsic drawback of poor morphology induced by the “coffee ring” effect,^[^ ^45^ ^]^ which will be discussed later.

**Figure 5:**
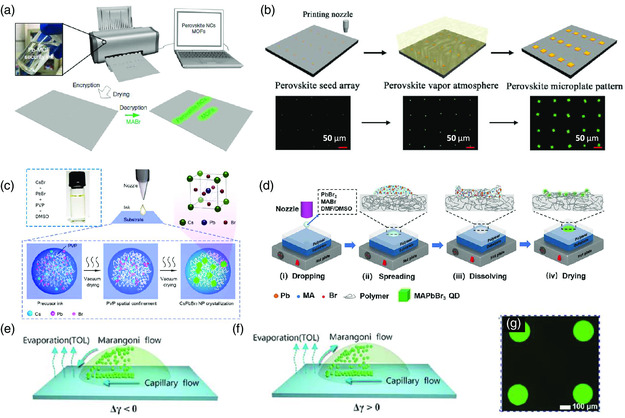
Inkjet printing of perovskite emitters. a) Schematic illustration of a two‐step inkjet printing, information encryption, and decryption process based on a lead–MOF recipe. Reproduced under the terms of the CC‐BY 4.0 license.^[^ ^46^ ^]^ Copyright 2017, The Authors, published by Springer Nature. b) Inkjet printing of controllable growth of perovskite microplate arrays. Schematic illustration of the vapor‐phase growth of perovskite microplate arrays (top images). The corresponding photoluminescence (PL) images during the perovskite growth process (bottom image). Reproduced with permission.^[^ ^48^ ^]^ Copyright 2020, Wiley‐VCH. c) Schematic illustration of an in situ formed perovskite nanocrystal pattern through inkjet printing of a perovskite precursor solution consisting of CsPbBr_3_ and PVP. Reproduced with permission.^[^ ^49^ ^]^ Copyright 2019, American Chemical Society. d) Schematic diagram of the “swelling–deswelling” of in situ formation of perovskite nanocrystals. Reproduced with permission.^[^ ^53^ ^]^ Copyright 2019, Wiley‐VCH. e,f) Schematic illustration of Marangoni flow and capillary flow under two assumptions. The surface tension in the top area is lower than that in the edge area (e). The surface tension in the top area is larger than that in the edge area (f). g) A PL image of inkjet‐printed perovskite quantum dots to reveal the suppressed “coffee ring” effects. e–g) Reproduced with permission.^[^ ^55^ ^]^ Copyright 2020, Royal Society of Chemistry.

Then, we turn to discuss a more complicated system, that is, the printing of nonluminance precursor solutions. Both one‐step and two‐step methods were exploited for printing the precursor solutions.^[^ ^46^, ^47^ ^]^ For example, Zhang et al. developed a two‐step way to obtain luminescent perovskite nanocrystals using a halide salt to trigger a lead‐based metal organic framework (MOF) fabricated with the inkjet printing method.^[^ ^46^ ^]^ Interestingly, due to the inherent ionic structure, the luminescence of the formed perovskite nanocrystals can be erased and recovered by polar solvent impregnation and halide retreatment, respectively, which then offers a chance to be used in confidential information encryption and decryption applications (**Figure** **5**a). Directly printing perovskite precursors without any additive addition to target high‐quality perovskite patterns is difficult, which is mainly attributed to the remarkably different nucleation and crystallization processes compared to the spin‐coating one. To obtain emissive perovskite pixels using the one‐step route, Gu et al. reported an accurately controlled epitaxial growth to fabricate highly luminescent CsPbBr_3_ microplate arrays with uniform morphology as well as controlled size and location (Figure 5b).^[^ ^48^ ^]^ This effective strategy depends on suppressed intractable lattice mismatches and the random nucleation barrier due to CsPbBr_3_ seeds introduced on silicon substrates, which facilitate the epitaxial growth of CsPbBr_3_ with the assistance of a vapor‐phase growth strategy.

An alternative strategy to obtain highly emissive perovskite emitters through the inkjet printing methods is in situ formation of perovskite nanocrystals through the incorporation of additional additives, such as, polyvinylpyrrolidone (PVP),^[^ ^49^ ^]^ into the precursor solutions. These introduced additives have functionality in both tuning the printable properties of the inks and controlling the final morphology as well as annihilating the defects in the perovskite patterns. The interaction between the lead ions and the functional group (i.e., the C=O group) along with the spatial confinement of the introduced polymers is critical for the formation of uniform perovskite nanocrystals with high PLQE. However, it is also noted that a high polymer ratio in functional inks significantly increases the viscosity of perovskite inks, making them unsuitable for printing. The Ohnesorge number (Oh) was developed to estimate the jettability of inks,^[^ ^50^ ^]^ defined as

(2) $$\text{Oh}=\frac{\mu }{\sqrt{\gamma \rho \frac{D}{2}}}$$

where *μ*, *γ*, *ρ*, and *D* are the ink viscosity, surface tension, density, and inkjet nozzle diameter, respectively. It was demonstrated that only Oh values ranging between 0.1 and 1 can guarantee the good jettability of inks.^[^ ^51^ ^]^

Considering that the viscosity is relevant to the temperature, a higher temperature usually results in decreased viscosity.^[^ ^52^ ^]^ Accordingly, a possible protocol to overcome the aforementioned unprintability issue is to increase the temperature of the nozzle as well as the precursor solutions to modulate the viscosity of the precursor inks. Through incorporating PVP into perovskite precursor solutions and adjusting the nozzle temperature, highly emissive perovskite nanocrystals with uniform size (≈30 nm) distribution and smooth morphology were obtained,^[^ ^49^ ^]^ as shown in Figure 5c. Shi et al. also reported in situ formed perovskite nanocrystals through a “swelling–deswelling” strategy in which perovskite precursors in DMSO or DMF were printed onto preheated polymer substrates,^[^ ^53^ ^]^ such as, polymethyl methacrylate (PMMA), polystyrene (PS), and polyvinylidene fluoride (PVDF). The soluble properties of these polymer materials in DMSO and DMF solvents make the polymer substrates partially dissolve or swell when perovskite inks contact and spread on them, leaving space for the formation of perovskite nanocrystals inside the polymeric matrix with further drying (Figure 5d).

We need to point out that regardless of the material system, the inkjet printing methods might impose challenges on precisely controlling the thickness and morphology of the perovskite patterns because of the widely reported “coffee ring” effect.[^45^, ^54^] The “coffee ring” effect was believed to arise from the capillary flow of particles initiated by a diverging evaporating flux from the center to the periphery of a pinned droplet on a solid surface (Figure 5e). In this case, the “coffee ring” effect may be inhibited by preventing the pinning of the contact line, tuning the distribution of the capillary flow toward the contact line, and suppressing the solutes being moved to the droplet edge by the capillary flows.^[45c]^ Through mixing a high‐boiling‐point solvent of dodecane with a low‐boiling‐point one of toluene to disperse the perovskite quantum dots, the “coffee ring” effect was effectively suppressed, enabling the fabrication of perovskite microarrays with a uniform and flat surface^[^ ^55^ ^]^ (Figure 5g). The mechanism behind the suppressed “coffee ring” by mixing two solvents with different boiling points was explained as the formation of appropriate Marangoni flow, which shows great potential to balance the capillary flow and therefore eliminates the “coffee ring” effect (Figure 5f). For the in situ formation case, it is reported that a rational amount of additional additive in the precursor solutions increases the viscosity of the precursor solutions, as well as having the possibility to withstand the “coffee ring” effect due to the suppressed outward capillary.^[^ ^49^ ^]^

### 4.2 E‐jet Printing

**Figure 6:**
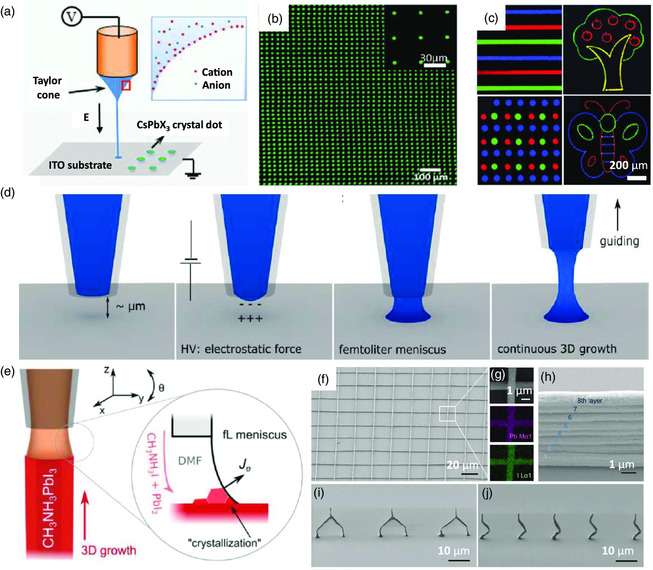
E‐jet printing of perovskite emitters. a) Schematic illustration of an e‐jet printing setup. b) A high‐resolution perovskite dot matrix with a diameter of ≈5 μm fabricated with the e‐jet printing method. c) Multicolor perovskite patterns fabricated with the e‐jet printing method. a–c) Reproduced with permission.^[56a]^ Copyright 2019, Wiley‐VCH. d) Schematic illustration of noncontact, meniscus‐on‐demand 3D e‐jet printing. Reproduced with permission.^[^ ^58^ ^]^ Copyright 2018, American Chemical Society. e) Schematic illustration showing meniscus‐guided 3D e‐jet printing of perovskite materials. f–j) Characterization of the different 3D structures of perovskites fabricated with the meniscus‐guided 3D e‐jet printing method. SEM images (f, h, i, j). EDX images (g). e–j) Reproduced with permission.^[^ ^59^ ^]^ Copyright 2019, Wiley‐VCH.

It is hard to deposit ultrahigh‐resolution patterns by using inkjet printing methods, which is attributed to the difficulty in the generation of an ultrasmall volume of droplets with widely used thermal and piezoelectric printer nozzles because of the extremely high levels of pressure required to overcome the capillary force in the printer nozzle.^[^ ^56^ ^]^ An alternative jet printing technique, that is, electrohydrodynamic jet (e‐jet) printing, provides an electric field–assisted way to achieve smaller droplets compared to inkjet printing.^[^ ^57^ ^]^ The e‐jet printing method relies on an electric field applied between the nozzle and the substrates (**Figure** **6**a), which initiates the flow of the ink from the nozzles to form an ultrasmall volume (i.e., femtoliter) of droplets. Inspired by the advantages of the e‐jet printing method, in situ formed CsPbX_3_ patterns with multiple‐color emission and high resolution with a pixel size of only 5 μm were obtained through using phenethylammonium bromide (PEABr) and 18‐crown‐6 to control the morphology and suppress the phase segregation between the perovskite and the organic compound of PEABr^[56a]^ (Figure 6b,c).

In addition, e‐jet printing offers the freedom of fabricating complex 3D microstructures over the inkjet printing methods, because the small volume of droplets allows the ink materials to directly crystallize during the printing process without the need of further posttreatment^[^ ^58^ ^]^ (Figure 6d). Chen and co‐workers demonstrated diverse perovskite nanostructures using a femtoliter meniscus of precursor inks, which was formed on an e‐jet printing head, to localize and guide solution‐mediated perovskite crystallization in midair^[^ ^59^ ^]^ (Figure 6e). In this case, MAPbI_3_ immediately started to grow inside the ink meniscus when the femtoliter‐volume ink was wetted on the substrate, forming the meniscus at the pipette–substrate gap. Consequently, this method promotes the fabrication of 3D perovskite nanostructures with high‐degree control over the printing diameter, direction, position, and even hollowness (Figure 6f–j), providing great potential to integrate the MHPs into a full‐color display and beyond.

### 4.3 Laser‐Assisted Patterning

Laser‐assisted patterning is another noncontact method for fabricating nanostructures on a large scale. It relies on photothermal heating, direct ablation, plasma formation, and photochemical reaction between a laser and active materials.^[^ ^60^ ^]^ For laser‐assisted patterning, the resolution of the patterns is decided to the diameter of the laser spot and the line‐width of the laser “pen.” In this part, we mainly discussed the photothermal heating and direction ablation branches because of their successful adoption for perovskite materials.

**Figure 7:**
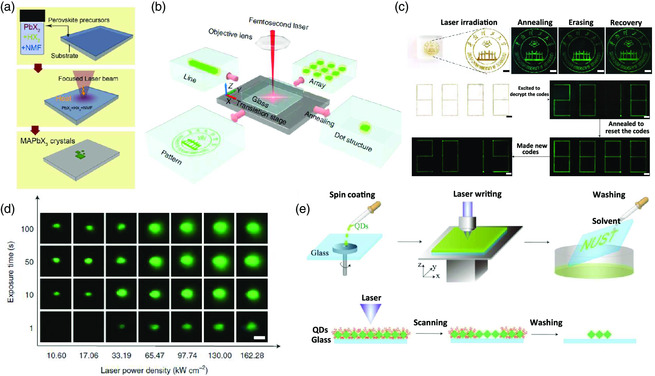
Laser‐assisted printing of perovskite emitters. a) Schematic illustration of laser‐induced formation of perovskite. Reproduced with permission.^[61b]^ Copyright 2017, Wiley‐VCH. b) Schematic of the femtosecond laser writing system for perovskite quantum dot fabrication. c) Optical and PL images of the laser‐induced formation of a CsPbBr_3_ quantum dot pattern after annealing, erasing, and recovery (scale bars: 500 μm). d) PL images of the femtosecond laser‐induced dots (after annealing) formed at different laser power densities and exposure times under 365 nm UV light (scale bar: 50 μm). b–d) Reproduced with permission.^[^ ^62^ ^]^ Copyright 2019, Springer Nature. e) Schematic illustration of laser ablation for patterning a perovskite quantum dot layer. Reproduced with permission.^[^ ^64^ ^]^ Copyright 2017, Wiley‐VCH.

Commonly, a conventional thermal annealing process is impossible to induce localized perovskite crystallization to form patterned perovskites. Fortunately, laser‐generated photothermal heating offers an alternative route to the limitation of localized perovskite crystallization, due to the controllable size, position, and light intensity of the laser pot.^[^ ^61^ ^]^ Arciniegas et al. reported an NIR laser as the energy source to induce perovskite formation from a thin wet precursor film consisting of PbX_2_ and hydrophilic acid in *N*‐methyl‐formamide (NMF).^[61b]^ Because the precursor films are NIR‐transparent, NIR light can be absorbed only by the bottom Si substrates, which generates localized thermal heating to trigger the formation of perovskites at specific sites on the substrate (**Figure** **7**a). Huang et al. also demonstrated a 3D laser printing method combined with a thermal treatment under high temperature to form perovskite quantum dots inside a transparent medium consisting of basic elements^[^ ^62^ ^]^ (Figure 7b). Intriguingly, the luminescent perovskite quantum dots in the transparent glass can be reversibly fabricated in situ and decomposed by repeating the laser irradiation and thermal annealing process (Figure 7c). In both the aforementioned cases, the properties of the laser (i.e., the laser intensity and laser exposure time) play an important role in determining the structure and quality of the perovskite patterns. A low laser power and short laser exposure time cannot induce the formation of perovskite nanocrystals due to insufficient energy accumulation. Enhancing the laser power or extending the laser exposure time leads to increased crystallization and diameter of the perovskite patterns (Figure 7d). However, it seems that the shapes of the laser‐assisted printed perovskite are not very uniform[^61^, ^62^] and need to be further improved for the fabrication of high‐resolution perovskite microarrays.

Instead of utilizing a laser to induce localized nucleation and crystallization, laser‐directed ablation via material decomposition is a simpler approach to control the fine structure and shape of perovskite patterns.^[^ ^63^ ^]^ Chen et al. reported a laser‐directed writing method for patterning perovskite quantum dot films.^[^ ^64^ ^]^ In this method, a continuous‐wave laser (405 nm) was used to write patterns on as‐deposited perovskite quantum dot films, where the surfactants on the laser‐irradiated area could be removed by laser irradiation (Figure 7e). As a result, the perovskite quantum dots at the rest area without laser exposure could be easily removed by rotational solvent washing, leaving the exposed quantum dots at the target substrates. However, the researchers also observed obvious PL reduction of the perovskite quantum dot film in both the laser‐writing and solvent‐washing steps. The degraded optical properties of the perovskite films should be attributed to the intrinsic instability of the perovskite materials under light exposure, the laser‐induced overheating, and the removal of capping ligands on the perovskite surface during solvent washing. To overcome the detrimental effects during laser ablation, Zhizhchenko and co‐workers demonstrated a chemically clean method of femtosecond laser ablation for MAPbI_3_ films, which reserves and to some extent improves the PL intensity of the perovskite films compared to the original one.^[^ ^65^ ^]^ The laser writing method provides a fast, simple, and programmable way to pattern perovskite materials without the drawback of the “coffee ring” effect as in ink‐printing techniques.

## 5 Mask‐Assisted Nanostructured MHPs

In spite of the facile fabrication process and simple equipment, the mask‐free methods may also raise concerns on precisely modulating the perovskite crystallization, film morphology, and shape of perovskite patterns. In industrial applications, mask‐assisted methods, such as, photolithography, UV/electron‐beam lithography, vacuum thermal evaporation, and template‐assisted transfer, are dominant for the fabrication of large‐area and ultrafine features. For the mask‐assisted methods, the morphology and structure of the patterned materials are relevant to the predesigned masks. In this section, we discuss three mask‐assisted strategies that have been adopted in LED applications, which are nanoimprinting, template‐induced patterning (photolithograph), and vacuum thermal evaporation.

### 5.1 Nanoimprinting

**Figure 8:**
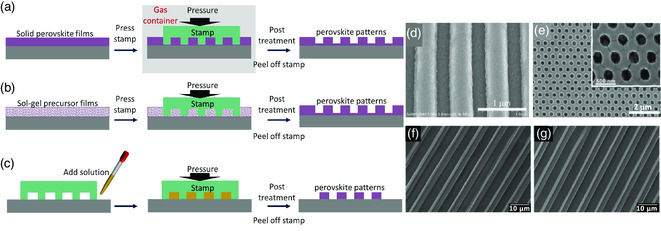
Nanoimprinting fabrication of patterned perovskite emitters. a–c) Schematic illustrations of nanoimprinting methods for perovskite films. Vapor‐assisted nanoimprinting for a solid‐state perovskite film (a). Nanoimprinting for a sol–gel perovskite precursor film (b). MIMIC method (c). d) Top‐view SEM image of MA‐assisted nanoimprinted perovskite films. d) Reproduced with permission.^[69a]^ Copyright 2017, Wiley‐VCH. e) Top‐view SEM image of perovskite pattern nanoimprinting from a sol–gel precursor film. Reproduced with permission.^[69b]^ Copyright 2017, Wiley‐VCH. f,g) Top‐view SEM image of perovskite pattern nanoimprinting from a sol–gel precursor film (f) and the MIMIC method (g). f,g) Reproduced with permission.^[^ ^70^ ^]^ Copyright 2018, American Chemical Society.

Nanoimprinting is a top–down printing method that has been widely utilized for large‐area and high‐throughput fabrication in industrial applications.^[^ ^66^ ^]^ In LED communities, nanoimprinting techniques were applied to pattern both of the emissive layers and other functional layers, such as, charge transport layers, to improve the performance of LEDs by extracting more light from devices.^[^ ^24^, ^67^ ^]^ The nanoimprinting strategies rely on a mold stamp with inverse patterns usually manufactured by electron‐beam lithography, photolithography, and etching techniques. For the nanoimprinting method, soft films, that is, polymer films, are desirable due to their easy deformation properties with suitable pressure upon the stamp and/or with thermal treatment.[^66^, ^68^] However, the hardness of most MHPs in a solid state makes directly nanoimprint the MHPs difficult. Thus, a growth or regrowth process of perovskites along with the predesigned mold is a key step to achieve perovskite patterns.^[^ ^69^ ^]^ According to the different procedures, the nanoimprinting methods can be further divided into vapor‐assisted regrowth of solid‐state perovskite films and template‐confined growth of precursor films or solutions, as shown in **Figure** **8**a–c, respectively. For the former one, taking MAPbI_3_ as an example, a solid‐state MAPbI_3_ film is first prepared with a common spin‐coating method, followed by thermal annealing treatment. Then, the predesigned mold stamp is placed on the obtained solid perovskite film and the whole sample is moved into an MA gas‐filled container to induce decomposition and recrystallization of the MAPbI_3_, and thus finally form patterned structures^[69a]^ (Figure 8a). Nevertheless, this gas‐assisted regrowth method has an obvious limitation that is not suitable for the fabrication of all‐inorganic MHPs—the inorganic metal salts are nonvolatile.

Alternatively, the template‐confined perovskite growth is not limited by the perovskite composition, because the predesigned soft or rigid stamps are usually used to confine the growth of a wet precursor film. As such, a precursor film with a rational amount of solvents remaining in it to form a sol–gel state is of great importance for template‐confined nanoimprinting. In detail, with the optimal pressure upon the stamp, the perovskite precursor films deform, along with the templates, followed by crystallization and the formation of perovskite crystals after carefully controlling the evaporation of the residual solvents. Then, patterned perovskite films with decent PL can be obtained after peeling off the mold stamps. In parallel to the nanoimprinting of solid and sol–gel state films, a micromolding capillaries (MIMIC) method was also exploited to nanoimprint precursor solutions.^[^ ^70^, ^71^ ^]^ In this method, a predesigned mold stamp was directly placed on a clean substrate to form capillaries between the stamp and the substrate, which introduce a driving force to transfer the perovskite precursor solutions from one end of the microchannel to the other end (Figure 8c). Again, after the solvent completely volatilized, the stamp was peeled off, leaving a patterned perovskite on the target substrates. The three aforementioned nanoimprinting strategies have been successfully adopted to prepare perovskite patterns with good optical properties and crystallization similar to the spin‐coated ones. However, as shown in the schematic diagram in Figure 8a–c, only the MIMIC method enables the formation of isolated perovskite patterns on a substrate, whereas the other approaches still leave some perovskite residuals in the rest area of the substrate.

Although nanoimprinting provides a simple procedure, and the reusability of the mold stamp may lower the fabrication cost, it also leaves unsolved problems for the fabrication of perovskite patterns with high quality. For example, as mentioned previously, the nucleation and crystallization behaviors of perovskite films depend on the degree of supersaturation of the perovskite solutions and interfacial energies. A mold stamp substrate covering the perovskite films or precursor films could make the nucleation and crystallization processes quite different. In addition, pressure on the stamp is inevitably needed to stress the deformation of the underlying perovskite materials mechanically; however, any inhomogeneous pressure distribution on the stamps will raise a challenge to achieving perovskite patterns with high uniformity. One may also need to note that it is still uncertain whether the nanoimprinting methods can be applied to pattern a red–green–blue perovskite matrix side by side at a high spatial resolution because it is still challenging to deposit perovskite films with multicolor emission on the same substrate through the commonly used spin‐coating, roll‐to‐roll, and blade‐coating methods.

### 5.2 Template‐Induced Patterning by Photolithography

**Figure 9:**
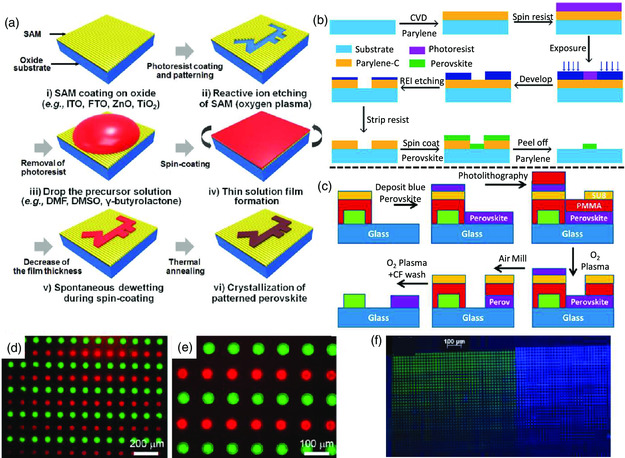
Photolithography fabrication of patterned perovskite emitters. a) Schematic illustration of wettability‐guided screen printing. b) Schematic illustration of bottom‐up photolithography fabrication of perovskite patterns. c) Schematic illustration of top–down photolithography fabrication of perovskite patterns. d,e) PL images of bottom‐up method fabricated multicolor perovskite patterns on the same substrate. f) PL images of top–down method fabricated multicolor perovskite patterns on the same substrate. a) Reproduced with permission.^[72d]^ Copyright 2020, Wiley‐VCH. b,d,e) Reproduced with permission.^[72b]^ Copyright 2020, American Chemical Society. c,f) Reproduced with permission.^[72c]^ Copyright 2019, American Chemical Society.

Compared to nanoimprinting, template‐induced patterning, especially photolithography and deep UV/electron‐beam lithography, may be available to fabricate high‐resolution perovskite patterns with high throughput. The template‐induced patterning of photolithography can mainly be categorized into the bottom‐up and top–down protocols.^[^ ^72^ ^]^ For the former one, the key step is using a lithographic method to produce a groove template for penetration of the precursor solutions. A simple bottom‐up template induced patterning is the wettability‐guided screen‐printing technique, as shown in **Figure** **9**a.^[72d]^ An electron‐beam lithography developed surface energy pattern was used to act as the screen printing template. Benefiting from the inherent properties of the wetting/dewetting behaviors of the precursor solutions, it can be only wetted in the groove‐template area but not on the rest area, thus guaranteeing the formation of high‐density crystal nuclei that promote the film densification with the target nanostructure. Nevertheless, this patterning technique only allows the fabrication of perovskite emitters with one emission color on the same substrate. More complicated bottom‐up or top–down strategies offer promising means to circumvent the aforementioned limitations, as shown in Figure 9b,c. In both methods, a photoresist and intermediary layer are crucial to achieve high‐quality perovskite patterns. Unlike the bottom‐up method using photolithography to generate a groove template for perovskite formation from a precursor solution, the top–down one utilizes photolithography to ablate solid perovskite films and then obtain target perovskite nanostructures.[^72^, ^73^] To obtain neat perovskite films on a target substrate, either the intermediary or the photoresist needs to be peeled off in both the bottom‐up and top–down methods, making it challenging to retain the initial optical properties of the perovskite pattern. This is possibly ascribed to the intrinsic instability of the perovskite materials, making it hard to find strict orthogonal solvents and resists to address the incompatibility of perovskites with the solvents used for photoresist and/or intermediary removal.

A dry peel‐off process, which relies on the weak adhesion between perylene‐C and the target substrates, was reported to safely remove the photoresist for the bottom‐up route.^[72b]^ In other words, the photoresist can be easily peeled off without using any solvent‐involved process, thus allowing repeating of the photolithographic process without damaging the former produced one. Consequently, multicolor perovskite emitters of green and red pixels together on the same substrate with a PLQE of over 60% can be achieved (Figure 9d,e). In addition, through utilizing a negative epoxy‐based resist of SU‐8, a top–down strategy for patterning high‐quality perovskite films was also successfully demonstrated.^[72c]^ In this method, an intermediary PMMA layer was introduced. The PMMA layer is ineffective as a photoresist in this top–down method; however, it can act as an easily removable intermediary layer to protect perovskite films from the solvents that are utilized to remove the photoresist of SU‐8, which enables the process of normal photolithography and achieves multicolor perovskite patterns on the same substrate (Figure 9f). The researchers highlighted this unique method for various perovskite materials, including both 3D and low‐dimensional perovskites, and it also works well with electron‐beam lithography for the fabrication of perovskite patterns as small as ≈1 μm. Yet, it also noted that the initial optical properties of the perovskite films will be influenced during the top–down photolithography process, as evidenced by the decreased PLQE of the patterned films compared to the fresh one.

### 5.3 Vacuum Thermal Evaporation

**Figure 10:**
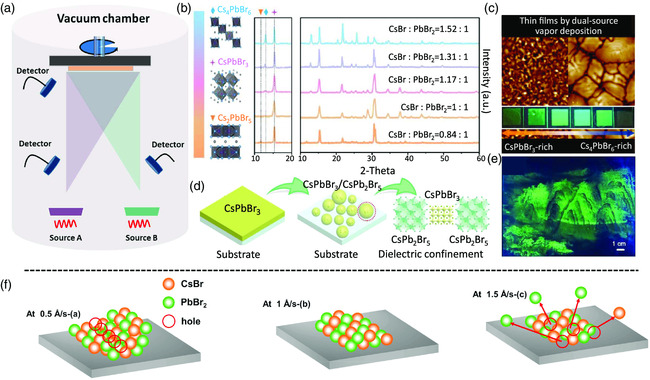
Vacuum thermally evaporated perovskite emitters. a) Schematic illustration of a setup for vacuum thermal evaporation. b) XRD patterns of vacuum thermally evaporated perovskite emitters with a different stoichiometric ratio between CsBr and PbBr_2_. Reproduced with permission.^[81b]^ Copyright 2019, Wiley‐VCH. c) Atomic force microscopy images and PL images of thermally evaporated perovskite emitters with a different stoichiometric ratio between CsBr and PbBr_2_. Reproduced with permission.^[^ ^82^ ^]^ Copyright 2017, American Chemical Society. d) Schematic illustration of perovskite transformation from CsPbBr_3_ to CsPb_2_Br_5_ and the dielectric confinement effect. e) A Chinese traditional landscape painting picture based on vacuum thermally evaporated perovskites. d,e) Reproduced with permission.^[^ ^84^ ^]^ Copyright 2019, Wiley‐VCH. f) Schematic illustration of the correlation between the evaporation rate influence and quality of the perovskite films. Reproduced with permission.^[81c]^ Copyright 2020, American Chemical Society.

All‐inorganic MHPs, such as, CsPbX_3_, display improved stability over organic–inorganic hybrid ones, in particular high thermal stability.[^17^, ^74^] In addition to the lead‐based perovskites, lead‐free derivatives, such as, Cs_3_Cu_2_I_5_^[^ ^75^ ^]^ and CsCu_2_I_3_,^[^ ^76^ ^]^ have also been developed for PeLED applications. However, the fast crystallization process and the limited solubility of both the all‐inorganic perovskites and their derivatives in the widely used solvents DMF and DMSO^[^ ^77^ ^]^ set obstacles to fabricating perovskite patterns with desirable film morphology using the aforementioned jet printing and photolithography methods. The vacuum thermal deposition method is an ideal choice that is not limited to the low solubility of the perovskites. Vacuum thermal deposition has been established as the most dominant method for industrial fabrication of OLED display panels.^[^ ^78^ ^]^ Therefore, the mature evaporating equipment and facilities may be facilely adapted to produce PeLEDs. In addition, the vacuum thermal evaporation method is not limited to the mechanical properties of the target substrates, enabling the deposition of perovskite films on soft, flexible, and even rough substrates. **Figure** **10**a shows the schematic illustration of the thermal vacuum deposition setup, in which two individual sources of AX and BX_2_ are coevaporated in a vacuum chamber. Vacuum thermal deposition of perovskite films in photovoltaic applications dates back to 2014,^[^ ^79^ ^]^ and now the power conversion efficiencies of vacuum thermally deposited perovskite solar cells are almost comparable to those of the solution‐processed ones.^[^ ^80^ ^]^ Notably, one may need to keep in mind that the state‐of‐the‐art perovskite emitters have remarkably different compositions in contrast to the active absorbers in photovoltaics, mainly due to the requirement of enhanced quantum and/or dielectric confinement effects as well as the high charge carrier transporting ability of the perovskite emitters in PeLEDs.[^1^, ^9^, ^34^] The existing studies of vacuum thermal evaporation systems mostly focus on engineering the quality of the perovskite films to improve the performance of PeLEDs,^[^ ^81^ ^]^ rather than developing high‐quality perovskite patterns. We believe that patterned perovskite pixels with fine‐feature shadow masks should have a negligible difference to large‐area films fabricated with or without large‐aperture shadow masks. Herein, we will discuss the existing strategies to obtain highly emissive perovskite films by tailoring the film composition as well as the crystallization kinetics.

First, the stoichiometric ratio of each initial component plays a crucial role in determining the crystallinity and photophysics of the deposited perovskite films. Taking all‐inorganic CsPbX_3_ as an example, an excess amount of CsX usually leads to enhanced crystallinity and PLQE, partially thanks to the formation of the Cs_4_PbBr_6_ phase[^81^, ^82^] (Figure 10b,c), which is similar to the solution‐processed films.^[^ ^83^ ^]^ In addition, in our previous work, we also demonstrated a vacuum thermally evaporated lead bromide–rich composite of CsPbBr_3_/CsPb_2_Br_5_ to target reversible PL with the assistance of air exposure and annealing treatments.^[^ ^84^ ^]^ Despite the decent optical features, the improvement in PL efficiency with the existence of parasites of either Cs_4_PbBr_6_ or CsPb_2_Br_5_ is still under debate.^[^ ^85^ ^]^ It was reported that both Cs4PbBr6 and CsPb2Br5 are nonfluorescent materials having a wide bandgap.^[^ ^86^ ^]^ Accordingly, some researchers believed that the possible mechanism for the PL enhancement should be attributed to passivation of grain boundaries, the formation of a host‐guest system,^[^ ^82^ ^]^ and the enhancement of the dielectric confinement effects^[^ ^84^ ^]^ with the existence of Cs4PbBr6 and CsPb2Br5 phases (Figure 10d). Meanwhile, researchers also pointed out that some intrinsic point defects in Cs_4_PbBr_6_ and CsPb_2_Br_5_ can also donate bright green PL,^[^ ^87^ ^]^ thus improving the optical properties of the obtained perovskite materials. Regardless of the uncertain mechanism beyond the improvement in the optical characterizations, it has been widely demonstrated that the formation of Cs_4_PbBr_6_ and CsPb_2_Br_5_ indeed helps to improve the PLQE of CsPbBr_3_; however, it may scarify the charge transporting ability simultaneously, leading to inferior device performance of PeLEDs. It is noteworthy that the PL emission of the vacuum thermally evaporated films can remain for more than 1 year in ambient conditions without obvious PL degradation, indicating the superior stability of such all‐inorganic perovskite films (Figure 10e).

In addition to the stoichiometric ratio, the crystallinity and photophysics of the perovskite films were also related to the thermal treatment, which can be divided into a postthermal annealing following the vacuum deposition and an in situ one during the deposition. For the former one, higher temperatures over 100 °C for CsPbBr_3_ and 200 °C for CsPbI_3_ are generally needed to completely convert the nonperovskite phase into perovskite,^[^ ^88^ ^]^ which may restrict the fabrication of flexible display panels on the common transparent plastic substrate, such as, polyethylene terephthalate (PET). In the case of the latter one, highly crystallized perovskites can be obtained through controlling the temperature of the substrates during the thermal evaporation process, without needing a postthermal annealing treatment. A relatively lower heating temperature has been demonstrated to obtain CsPbBr_3_ films with high quality, which is favorable for the fabrication of flexible optoelectronics.^[81c]^ An optimal temperature not only influences the crystallinity and morphology of the deposited perovskite films but also determines the defects in the films. For example, obvious defects in the crystal grain were observed along the vertical direction of the film during an in situ thermal evaporation process at 30 °C, whereas the defects were obviously reduced in the 60 °C processed sample.

We also need to pay attention to the evaporation rate of each source, which influences the morphology and defects of the deposited perovskite films as well.[^81^, ^89^] Again, taking CsPbB_3_ as an example, a possible mechanism under different evaporation rates for perovskite film growth was proposed, as shown in Figure 10f. The researchers claimed that the different deposition rates of CsBr and PbBr_2_ dramatically affect the film‐forming kinetics and the final morphology of the deposited films.^[81c]^ In detail, when the perovskite films are deposited at a low evaporation rate, island‐like structures are likely to be formed, due to a more pronounced adhesion of CsBr molecules to the surface, which may generate defects in the films grown along with specific crystal growth. In contrast, a high evaporation rate may lead to collisions between large‐molecular‐weight molecules of PbBr_2_ or collisions with the existing film, generating voids in the deposited films. Only a medium evaporation rate can enable the fabrication of uniform layers because of the optimized homogeneous adhesion of CsBr and PbBr_2_ on the substrates.

## 6 Bring MHPs into Prototype of Full‐Color Displays

**Figure 11:**
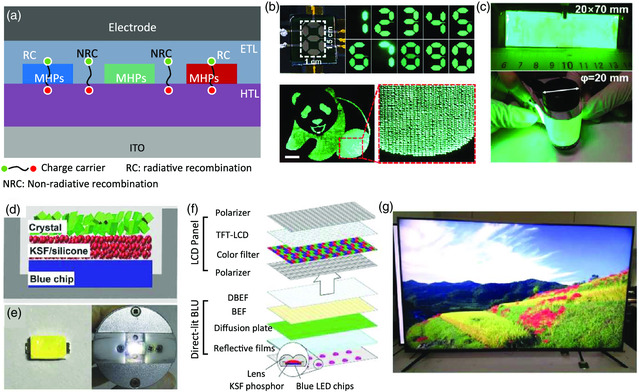
PeLEDs for full‐color display. a) Schematic illustration of the direct interfacial contact in matrix PeLEDs, which may result in undesirable nonradiative recombination loss. b) Applications of matrix PeLEDs in dynamic digital and a 200 pixel per in. (ppi) display (scale bar: 500 μm). Reproduced with permission.^[^ ^91^ ^]^ Copyright 2020, American Chemical Society. c) A green flexible PeLED fabricated by the vacuum thermal evaporation method before and after stretch. Reproduced with permission.^[81c]^ Copyright 2020, American Chemical Society. d) Schematic illustration of the structure of a white PeLED. e) A picture of the surface‐mounted device (left) and a working PeLED (right). d,e) Reproduced with permission.^[77b]^ Copyright 2018, Wiley‐VCH. f) Schematic diagram of PQDCF LCD TV prototype. g) A 55 in. full‐color TV display with perovskites with backlight units. f,g) Reproduced with permission.^[^ ^95^ ^]^ Copyright 2018, Wiley‐VCH.

We have reviewed several methods to obtain perovskite patterns earlier; however, their application in PeLEDs, especially electrically driven self‐emitting PeLED arrays, has been rarely reported. The recorded EQE of an inkjet‐printed green PeLED is only 2.2% with a brightness of about 1200 cd m^−2^, which is still far behind the device performance of a normal spin‐coated one.[^1^, ^90^] The low device performance of current inkjet‐printed PeLEDs may be attributed to three main reasons: the undesirable film morphologies; the trade‐off between the PLQE and charge transport ability of the perovskite films; and the poor interfacial contacts between each functional layer. Regarding the film morphologies, the detrimental “coffee ring” effect may substantially increase the nonradiative recombination loss in devices. Also, to obtain highly emissive emitters in these in situ formatted perovskite nanocrystal systems, the introduced additives (most of them are polymers) help to improve the PLQE but simultaneously scarify the charge transport ability and therefore reduce the charge injection efficiency. It is also noted that in these PeLEDs with patterned perovskites, only emissive layers have been fabricated with printing methods, whereas the other functional layers, that is, charge‐transporting layers and electrodes, have still been deposited by the widely used spin‐coating or thermal evaporation methods. Consequently, the top charge transporting layer and/or metal electrode might directly contact with the bottom ones through pinholes, cracks in perovskite films, or a blank area without perovskite coverage. Then, the injected charge carriers may directly pass through the perovskite emitters without any radiative recombination, leading to device shorting problems, as schematically shown in **Figure** **11**a. Therefore, patterning all the functional layers or introducing an insulating interfacial layer between the charge transporting layers and perovskite emitters may partially help to overcome the aforementioned problems. In spite of the low efficiencies of PeLEDs based on perovskite patterns, the feasibility of potential applications of a dynamic digit 7‐segment display and a static EL image with 200 dpi was successfully demonstrated,^[^ ^91^ ^]^ as shown in Figure 11b.

In terms of the interfacial contacts, the thermal vacuum deposition protocol is an ideal choice for the fabrication of matrix PeLEDs with improved device performance because each functional layer can be deposited in sequence with a predesigned shadow mask. We also point out that there is still no report on the fabrication of matrix PeLEDs with the vacuum thermal evaporation method, which is expected to be achieved in the near future by selecting suitable shadow masks. However, through tailoring the composition of the perovskite films, the annealing process, and the charge transport layers, PeLEDs with a vacuum thermally evaporated perovskite emissive layer exhibit an EQE of 3.26% and brightness of around 10 000 cd m^−2^.^[81c]^ In addition, this method shows great potential for the fabrication of large‐area flexible PeLEDs (Figure 11c). The key challenge to further improving the device performance is how to enhance the relatively low PLQE of the deposited perovskite films. Due to the absence of passivation molecules in the films, the PLQEs of most vacuum thermally evaporated perovskite films are still below 40%, much lower than those of most solution‐processed ones. In these high‐performance PeLEDs fabricated with spin‐coating methods, additional additives, such as, alkali metal halides,[^74^, ^92^] Lewis bases,[^1^, ^20^, ^93^] and ammonium halides,[^9^, ^14^, ^33^] are highly required for the annihilation of trap states by governing the crystallization process and/or passivating the dangling bonds as well as forming low‐dimensional structures, which have been rarely adopted in vacuum thermally evaporated perovskite films.

Self‐emitting PeLEDs exhibit marvelous merits such as simple device structure, low energy consumption, high color purity, and brightness contrast. In addition, integrating these high‐quality perovskite materials into LCD panels as pure color filters or emitters also shows great potential for the fabrication of high‐fidelity displays.[^36^, ^94^] A common way is to encapsulate the perovskite emitters onto a blue LED chip to partially replace the rare earth phosphors for white LED backlighting units (Figure 11d,e). For example, with the combination of green perovskite emitters and red K_2_SiF_6_:Mn_4_ on a blue LED chip, a prototype white PeLED with a high luminous efficiency of 151 lm W^−1^ and a color gamut of 90.6% of Rec. 2020 can be achieved.^[77b]^ All the parameters are much better than those of conventional LCD techniques. Inspired by the outstanding performance, a perovskite quantum dot embedded composite film (PQDCF) LCD TV prototype was designed and demonstrated,^[^ ^95^ ^]^ as shown in Figure 11f, in which the blue light emitted from the bottom LED chips was partially converted into red and green light by the top phosphor and perovskite materials. Later, a Chinese display company, TCL, launched the first perovskite emitter–involved 55″ TV panel with a color gamut of 101% and a highest brightness of 500 nits^[^ ^95^ ^]^ (Figure 11g), which may bring a revolution in the field of displays in the foreseeable future.

## 7 Outlook

### 7.1 Achieving Perovskite Patterns with Improved Optical and Electrical Properties

In spite of the unique properties of perovskite materials, their intrinsic instability under thermal heating, UV light, and moisture conditions may impose challenges to achieving perovskite patterns with decent optical and electrical properties compared to the normal spin‐coated ones, especially for the fabrication of pure‐blue, ‐green, and ‐red perovskite microarrays side by side with high spatial resolution. In the previous sections, we have mentioned that the PL intensity of perovskite patterns may deteriorate during the laser writing or photolithography procedure. Thus, carefully controlling the patterning procedures to reserve the optical properties of the perovskite film is of fundamental importance to achieve high‐performance PeLEDs with microstructured emissive layers. For example, a photolithography process that is relatively harsh for perovskite materials should be optimized to suppress detrimental effects by depositing protective layers or avoiding any extra photoresist utilization. Regarding the vacuum thermal system, the inferior PLQEs of the deposited films are one of the main limitations for developing efficient PeLEDs. According to the well‐studied PeLEDs with spin‐coated methods, the PLQEs are mainly limited by the defect‐assisted nonradiative recombination loss in the perovskite films. Incorporating functional additives could remarkably improve the PLQEs and overall performance of the resulting PeLEDs. However, investigation on passivating the defects by incorporating these well‐studied additives into vacuum thermally evaporation perovskites is still lacking, possibly due to technical limitations. Alternatively, a posttreatment, that is, depositing a passivation layer on the perovskite films and gas‐assisted healing,^[^ ^96^ ^]^ may be a practical approach to improve the PLQE of thermal evaporation–deposited perovskite films.

The nucleation and crystallization kinetics also significantly determine the optical and electrical properties of perovskite patterns. For spin‐coated films, antisolvent engineering has been frequently exploited to modulate the nucleation and crystallization kinetics to improve the film morphology and/or reduce the nonradiative recombination centers. Nevertheless, for the jet printing, laser writing, nanoimprinting, and vacuum thermal evaporation methods, it will be difficult to utilize such a promising strategy to improve the photoelectronic features of perovskite patterns. It will be helpful to get a deep insight into the crystallization kinetics during the patterning stage by using advanced analytic techniques that are already well developed to investigate spin‐coated perovskite films. In this way, developing specialized protocols to control the crystallization process of perovskite patterns with outstanding optical and electrical properties will become possible.

### 7.2 Further Improving Device Performance of PeLEDs

A full‐color display requires high EL efficiency in all three primary colors of pure‐blue, ‐green, and ‐red emission. At present, the EQEs of pure‐red and ‐green PeLEDs have surged to over 20%,[^1^, ^5^] whereas the recorded pure‐blue one is still below 10%.^[^ ^14^, ^97^ ^]^ Therefore, further enhancing the performance of pure‐blue PeLEDs is an urgent task for full‐color displays. The common strategies to achieve pure‐blue emission can be categorized into three technical directions, that is, mixing halides,^[^ ^98^ ^]^ reducing dimensionality,^[^ ^4^, ^14^, ^99^ ^]^ and a combination of these.^[^ ^100^ ^]^ However, both of these have their drawbacks for achieving efficient and stable pure‐blue PeLEDs. For the halide‐mixed one, a core problem is the easy phase segregation between perovskites with different halide components, which can result in degraded device performance and irreversible EL shift during bias measurement. In the case of the low‐dimensional one, the broad emission width induced by the coexistence of perovskite nanoplatelets with different *n* values may scarify the color purity of PeLEDs. Furthermore, the low charge transport ability, ascribed to the insulation properties of the large surface ligands, also compromises the performance of the PeLEDs. How to suppress the phase segregation in halide‐mixed perovskites and control the *n* distribution as well as the charge transport ability in low‐dimensional perovskites requires extensive investigations to further improve the performance of pure‐blue PeLEDs.

The other issue that may restrict the practical application of PeLEDs is the poor operational stability, especially of the blue ones, which only survive several minutes even measured under a very low luminescence.^[^ ^4^, ^100^ ^]^ As evidenced in perovskite photovoltaics, it is believed that the operational stability of PeLEDs is highly correlated to the ion migration of halide and electrode ions, the quality of interfacial contacts between the charge transport layers and perovskite active layers, and the intrinsic instability of perovskite active materials.^[^ ^34^, ^101^ ^]^ We suppose that the degradation mechanism beyond the poor operational stability of PeLEDs is more complicated than that of the photovoltaic devices due to the harsh working conditions, that is, higher electrical fields and working temperatures. Therefore, some successful strategies adopted to improve the stability of perovskite photovoltaics may not work well in PeLEDs. However, it is still believed that by further suppressing the ion migration by reducing the trap defects (ion migration channels), balancing the charge injection, and building more robust interfacial contacts, the operational stability of PeLEDs may catch up with that of OLEDs and QLEDs soon.

### 7.3 Developing Eco‐Friendly PeLEDs

The heavy metal element of lead in most perovskites may raise the concern of their toxicity,^[^ ^102^ ^]^ even though the total amount of Pb^2+^ ions should be extremely low in a real PeLED display panel. A wide range of lead‐free perovskites and their derivatives have been developed, such as Sn‐based perovskites[^2^, ^103^] and “double perovskites”;[^94^, ^104^] however, the overall performance of these lead‐free PeLEDs is still lagging far behind. In addition, the Sn‐based PeLEDs are easily oxidized from Sn^2+^ to Sn^4+^, which deteriorates the perovskite crystal structure and degrades the overall device performance. For “double perovskites” and their derivatives, it is still challenging to deposit smooth perovskite films targeting high‐performance PLEDs. Some “double perovskite” derivatives are indirect‐bandgap materials, which also hinders their applications in self‐emitting PeLEDs. Therefore, a combination of theoretical and experimental investigations on these lead‐free perovskites and their derivatives may be helpful to build a correlation between the morphological, optical, and electrical properties and the performance of lead‐free PeLEDs.

Aside from the toxic metal elements in perovskite materials, the solvents used to deposit both perovskites and other functional layers for PeLEDs may also burden the chronic health risks and fabrication costs. The widely used aromatic solvents of CB and polar solvents of DMF for fabricating charge transport and perovskite layers are not desirable for industrial applications because of their negative impact on human health and the environment.^[^ ^105^ ^]^ According to the well‐investigated perovskite photovoltaics, a lot of greener solvents, such as anisole,^[^ ^106^ ^]^ gamma butyrolactone (GBL),^[^ ^107^ ^]^ and tetrahydrofuran (THF), have been utilized to reduce the toxic threats. However, almost all the efficient PeLEDs utilized the toxic CB and DMF as solvents, which calls for more investigations on decreasing the chronic health risks, which is equally important for bringing eco‐friendly PeLEDs into our daily life.

## Conflict of Interest

The authors declare no conflict of interest.
